# Sense and Sensibility: Sensible Quality Control and Data Decision-Making in Passive Sensing Data for Adolescent Substance Use Risk Prediction

**DOI:** 10.64898/2026.10.06.26364884

**Published:** 2026-10-08

**Authors:** Anni Hong, Jihui L. Diaz, Traci M. Kennedy, Frances L. Wang

## Abstract

**Background:**

Passively collected smartphone and wearable data, combined with genomic information, may enable scalable early identification of youth who are at risk for substance use disorder. However, heterogeneous data collected in naturalistic settings limit researcher control over data quality, requiring substantial preprocessing. Data cleaning and feature engineering decisions can meaningfully influence analytic outcomes yet are often minimally described or omitted in published work.

**Objectives:**

We provide rationales and recommendations for common preprocessing challenges in passive sensing research, including establishing inclusion criteria when “outliers” may reflect true behavioral variability, analyzing differences across smartphone operating systems, handling observations collected outside protocol windows, and engineering features for machine learning applications.

**Methods:**

We illustrate principled approaches to these challenges using smartphone and Fitbit data from the Adolescent Brain Cognitive Development ^SM^ Study (ABCD). The sample was drawn from the available half of the year-4 follow-up (data release 5.1; N = 4,754; ages 13–14).

**Results:**

Extreme outlying observations were concentrated among low-engagement participants with few observed days. Smartphone usage among Apple users appeared under-recorded relative to Android users when compared with self-report measures. Fitbit-derived behavior differed systematically between observations collected within versus outside protocol windows. We also identified potential errors in the study’s default preprocessing pipeline.

**Conclusion:**

We recommend prioritizing person-level inclusion criteria that preserve meaningful behavioral variability, examining temporal distributions rather than assuming observations are exchangeable, and thoroughly investigating and reporting data quality issues. Although illustrated using the ABCD Study, these principles broadly apply to research using smartphones, wearables, and other digital data sources.

## Full Text Availability

The license terms selected by the author(s) for this preprint version do not permit archiving in PMC. The full text is available from the preprint server.

